# Mesaconitine

**DOI:** 10.1107/S1600536808013147

**Published:** 2008-05-10

**Authors:** Dao-Hang He, Yong-Chuang Zhu, Ai-Xi Hu

**Affiliations:** a School of Chemistry and Chemical Engineering, South China University of Technology, Guangzhou 510641, People’s Republic of China; b College of Chemistry and Chemical Engineering, Hunan University of Technology, Changsha 410082, People’s Republic of China

## Abstract

The title compound, (1α,3α,6α,14α,15α,16β)-3,8,13,14,15-penta­hydr­oxy-1,6,16-trimeth­oxy-4-methoxy­methyl-20-methyl­acon­itan-8,14-diyl 8-acetate 14-benzoate, C_33_H_45_NO_11_, a C_19_ diterpenoid alkaloid, obtained from the roots of *Aconitum kusnezoffii*, has been crystallographically characterized in this study. Rings *A*, *B* and *E* have chair conformations, rings *C* and *F* display envelope conformations, and ring *D* adopts a boat conformation. There are inter- and intra­molecular O—H⋯O hydrogen bonds, the latter resulting in the formation of a non-planar seven-membered ring. The inter­molecular inter­actions link the mol­ecules into a two-dimensional network.

## Related literature

For general background, see: Hikino *et al.* (1980 ▶); Li *et al.* (1997 ▶); Mitamura *et al.* (2002 ▶); Saito *et al.* (1982 ▶); For ring conformation details, see: Codding (1982 ▶); De Camp & Pelletier (1977 ▶); Parvez *et al.* (1999 ▶); Pelletier *et al.* (1982 ▶). For related literature, see: Pelletier & Djarmati (1976 ▶); Tsuda & Marion (1963 ▶); Zhapova *et al.* (1986 ▶).
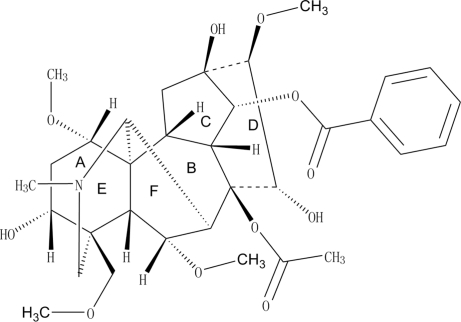


## Experimental

### 

#### Crystal data

C_33_H_45_NO_11_
*M* *_r_* = 631.70
Orthorhombic, 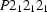

*a* = 12.6820 (6) Å
*b* = 15.3848 (7) Å
*c* = 15.6110 (7) Å
*V* = 3045.9 (2) Å^3^
*Z* = 4
Mo *K*α radiation
μ = 0.10 mm^−1^
*T* = 173 (2) K
0.46 × 0.35 × 0.12 mm

#### Data collection

Bruker SMART 1000 CCD diffractometer
Absorption correction: multi-scan (*SADABS*; Sheldrick, 2003 ▶) *T*_min_ = 0.954, *T*_max_ = 0.988
18319 measured reflections
3713 independent reflections
3026 reflections with *I* > 2σ(*I*)
*R*_int_ = 0.042

#### Refinement

*R*[*F* ^2^ > 2σ(*F* ^2^)] = 0.038
*wR*(*F* ^2^) = 0.081
*S* = 1.07
3713 reflections
418 parameters
2 restraints
H atoms treated by a mixture of independent and constrained refinement
Δρ_max_ = 0.21 e Å^−3^
Δρ_min_ = −0.22 e Å^−3^

### 

Data collection: *SMART* (Bruker, 2001 ▶); cell refinement: *SAINT-Plus* (Bruker, 2003 ▶); data reduction: *SAINT-Plus*; program(s) used to solve structure: *SHELXTL* (Sheldrick, 2008 ▶); program(s) used to refine structure: *SHELXTL*; molecular graphics: *SHELXTL*; software used to prepare material for publication: *SHELXTL*.

## Supplementary Material

Crystal structure: contains datablocks I, global. DOI: 10.1107/S1600536808013147/wn2256sup1.cif

Structure factors: contains datablocks I. DOI: 10.1107/S1600536808013147/wn2256Isup2.hkl

Additional supplementary materials:  crystallographic information; 3D view; checkCIF report

## Figures and Tables

**Table 1:** Hydrogen-bond geometry (Å, °)

*D*—H⋯*A*	*D*—H	H⋯*A*	*D*⋯*A*	*D*—H⋯*A*
O10—H10*A*⋯O2	0.84	2.11	2.788 (3)	138
O7—H7⋯O8	0.84	2.04	2.560 (3)	120
O4—H4⋯O11^i^	0.84	2.20	3.018 (3)	163

Symmetry code: (i) 
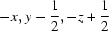
.

**Fig. 1.**
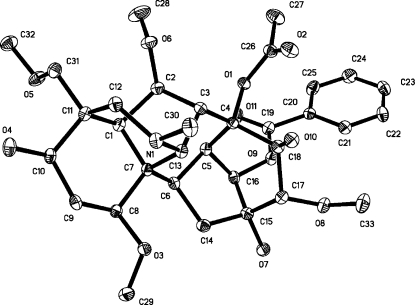
A view of the structure of the title compound, showing the atom-numbering scheme. Displacement ellipsoids are drawn at the 30% probability level. H atoms have been omitted for clarity.

**Fig. 2.**
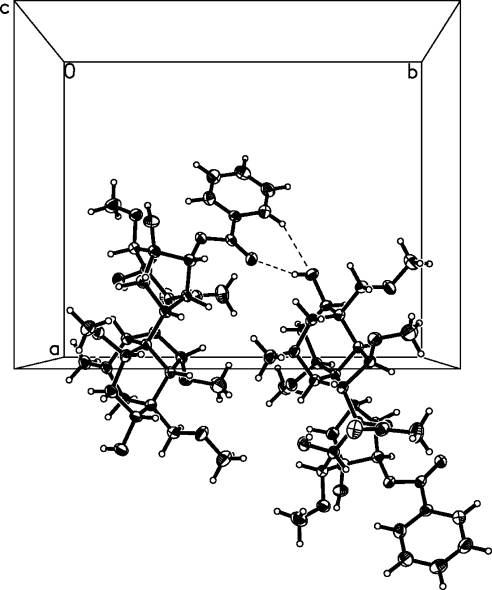
The packing of molecules of the title compound, viewed down the c axis. Dashed lines indicate hydrogen bonds.

## supplementary crystallographic information

### Comment

As an important Chinese herbal medicine belonging to the genus *Aconitum*, *A. kusnzoffii* has been therapeutically used to treat rheumatic pain, paralysis due to stroke, rheumatoid arthritis and some other inflammations. Mesaconitine, a C_19_ diterpenoid alkaloid, is pharmacologically one of the most active components obtained from the roots of *A. kusnezoffii* (Li *et al.*, 1997). Mesaconitine has been reported to have an anti-inflammatory activity (Hikino *et al.*,1980; Saito *et al.*, 1982), and the vasorelaxant effect of mesaconitine may contribute to the therapeutical effectiveness on persons with a weak constitution and poor metabolism, by improving peripheral blood circulation (Mitamura *et al.*, 2002). The three-dimensional structure of most biologically active molecules plays a role in governing their interactions and activities. It is important to obtain information on the mode of action and selectivity of mesaconitine so that it can be used safely and efficiently. Many X-ray crystal structure determinations of C_19_ diterpenoid alkaloids have been reported, such as pseudaconitine, delphinine (Parvez *et al.*, 1999; Pelletier *et al.*, 1982.). However, the crystal structure of mesaconitine has not been reported. In view of this, the crystal structure determination of the title compound was carried out and the results are presented here.

The structure of mesaconitine is similar to that of aconitine (Codding, 1982). The only difference between aconitine and mesaconitine is a methylene group at the tertiary nitrogen atom. The bond lengths and angles in the title compound are in good agreement with expected values. In the molecule of the title compound, (Fig. 1), rings A, B and E have a chair conformation, rings C and F display an envelope conformation, ring D adopts a boat conformation. The packing of the title compound is shown in Fig. 2. In the crystal structure, there are inter- and intramolecular O—H···O hydrogen bonds. The former link the molecules into a two-dimensional network, while the latter results in the formation of a non-planar seven-membered ring. These intramolecular hydrogen bonds may be effective in the stabilization of the structure.

### Experimental

The title compound was isolated from the roots of *A. kusnezoffii* according to the literature procedure of Li *et al.* (1997) and crystals of X-ray quality were grown from methanol at room temperature by slow evaporation.

### Refinement

The H atom attached to C2 was located and refined freely [C—H = 0.97 (2) Å]. Other H atoms were included in the refinement at idealized positions and refin ed as riding, with C—H = 0.95 (aromatic), 0.98 (CH_2_), 1.00 (CH), O—H = 0.84 Å. U_iso_(H) = xU_eq_(carrier atom), where x = 1.5 for O and methyl, 1.2 for all other H atoms. In the absence of significant anomalous scattering effects, Friedel pairs were merged. The absolute configuration was assigned on the basis of the related literature (Pelletier & Djarmati, 1976; Tsuda & Marion, 1963; Zhapova *et al.*, 1986).

### Crystal data

**Table:**

C_33_H_45_NO_11_	*F*_000_ = 1352
*M**_r_* = 631.70	*D*_x_ = 1.378 Mg m^−^^3^
Orthorhombic, *P*2_1_2_1_2_1_	Mo *K*α radiation λ = 0.71073 Å
Hall symbol: P 2ac 2ab	Cell parameters from 6314 reflections
*a* = 12.6820 (6) Å	θ = 2.5–26.8º
*b* = 15.3848 (7) Å	µ = 0.10 mm^−^^1^
*c* = 15.6110 (7) Å	*T* = 173 (2) K
*V* = 3045.9 (2) Å^3^	Block, colorless
*Z* = 4	0.46 × 0.35 × 0.12 mm

### Data collection

**Table:**

Bruker SMART 1000 CCD diffractometer	3713 independent reflections
Radiation source: fine-focus sealed tube	3026 reflections with *I* > 2σ(*I*)
Monochromator: graphite	*R*_int_ = 0.042
*T* = 173(2) K	θ_max_ = 27.0º
ω scans	θ_min_ = 1.9º
Absorption correction: multi-scan(SADABS; Sheldrick, 2003)	*h* = −16→15
*T*_min_ = 0.954, *T*_max_ = 0.988	*k* = −16→19
18319 measured reflections	*l* = −16→19

### Refinement

**Table:**

Refinement on *F*^2^	Secondary atom site location: difference Fourier map
Least-squares matrix: full	Hydrogen site location: inferred from neighbouring sites
*R*[*F*^2^ > 2σ(*F*^2^)] = 0.038	H atoms treated by a mixture of independent and constrained refinement
*wR*(*F*^2^) = 0.081	*w* = 1/[σ^2^(*F*_o_^2^) + (0.0245*P*)^2^ + 1.2755*P*] where *P* = (*F*_o_^2^ + 2*F*_c_^2^)/3
*S* = 1.07	(Δ/σ)_max_ = 0.001
3713 reflections	Δρ_max_ = 0.21 e Å^−^^3^
418 parameters	Δρ_min_ = −0.22 e Å^−^^3^
2 restraints	Extinction correction: none
Primary atom site location: structure-invariant direct methods	

### Special details

**Table:**

Geometry. All e.s.d.'s (except the e.s.d. in the dihedral angle between two l.s. planes) are estimated using the full covariance matrix. The cell e.s.d.'s are taken into account individually in the estimation of e.s.d.'s in distances, angles and torsion angles; correlations between e.s.d.'s in cell parameters are only used when they are defined by crystal symmetry. An approximate (isotropic) treatment of cell e.s.d.'s is used for estimating e.s.d.'s involving l.s. planes.
Refinement. Refinement of *F*^2^ against ALL reflections. The weighted *R*-factor *wR* and goodness of fit *S* are based on *F*^2^, conventional *R*-factors *R* are based on *F*, with *F* set to zero for negative *F*^2^. The threshold expression of *F*^2^ > σ(*F*^2^) is used only for calculating *R*-factors(gt) *etc*. and is not relevant to the choice of reflections for refinement. *R*-factors based on *F*^2^ are statistically about twice as large as those based on *F*, and *R*- factors based on ALL data will be even larger.

### Fractional atomic coordinates and isotropic or equivalent isotropic displacement parameters (Å^2^)

**Table:**

	*x*	*y*	*z*	*U*_iso_*/*U*_eq_	
C1	−0.0391 (2)	0.81198 (18)	0.21029 (19)	0.0220 (6)	
H1	−0.0477	0.8647	0.1734	0.026*	
C2	0.0052 (2)	0.8377 (2)	0.3007 (2)	0.0246 (7)	
C3	0.0859 (2)	0.76493 (18)	0.32284 (18)	0.0218 (6)	
H3	0.0727	0.7412	0.3815	0.026*	
C4	0.1968 (2)	0.80413 (18)	0.31663 (18)	0.0209 (6)	
C5	0.2045 (2)	0.85444 (18)	0.23165 (18)	0.0219 (6)	
H5	0.1701	0.9126	0.2373	0.026*	
C6	0.1544 (2)	0.80248 (19)	0.15592 (18)	0.0220 (6)	
H6	0.1380	0.8456	0.1099	0.026*	
C7	0.0505 (2)	0.75172 (18)	0.17449 (18)	0.0207 (6)	
C8	0.0175 (2)	0.70445 (19)	0.09137 (19)	0.0226 (6)	
H8	0.0235	0.7470	0.0432	0.027*	
C9	−0.0936 (2)	0.6672 (2)	0.0894 (2)	0.0256 (7)	
H9A	−0.1126	0.6534	0.0294	0.031*	
H9B	−0.0949	0.6123	0.1225	0.031*	
C10	−0.1744 (2)	0.72861 (19)	0.12591 (19)	0.0241 (6)	
H10	−0.1762	0.7807	0.0877	0.029*	
C11	−0.1439 (2)	0.76086 (19)	0.21596 (19)	0.0236 (6)	
C12	−0.1281 (2)	0.68527 (19)	0.2794 (2)	0.0254 (7)	
H12A	−0.1312	0.7082	0.3386	0.030*	
H12B	−0.1866	0.6432	0.2724	0.030*	
C13	0.0663 (2)	0.69431 (18)	0.25371 (18)	0.0226 (6)	
H13	0.1306	0.6574	0.2466	0.027*	
C14	0.2486 (2)	0.7463 (2)	0.1230 (2)	0.0275 (7)	
H14A	0.2335	0.6837	0.1313	0.033*	
H14B	0.2606	0.7570	0.0612	0.033*	
C15	0.3455 (2)	0.7728 (2)	0.17473 (18)	0.0247 (6)	
C16	0.3180 (2)	0.86471 (19)	0.20170 (19)	0.0242 (6)	
H16	0.3214	0.9048	0.1513	0.029*	
C17	0.3584 (2)	0.71420 (19)	0.25396 (19)	0.0253 (7)	
H17	0.3397	0.6533	0.2376	0.030*	
C18	0.2910 (2)	0.74092 (19)	0.33247 (18)	0.0240 (6)	
H18	0.3398	0.7717	0.3726	0.029*	
C19	0.3869 (2)	0.97097 (19)	0.3007 (2)	0.0257 (7)	
C20	0.4690 (2)	0.98015 (19)	0.3682 (2)	0.0250 (6)	
C21	0.5301 (3)	0.9093 (2)	0.3930 (2)	0.0299 (7)	
H21	0.5187	0.8540	0.3677	0.036*	
C22	0.6072 (3)	0.9198 (2)	0.4544 (2)	0.0356 (8)	
H22	0.6489	0.8715	0.4713	0.043*	
C23	0.6240 (3)	0.9998 (2)	0.4915 (2)	0.0365 (8)	
H23	0.6767	1.0065	0.5342	0.044*	
C24	0.5643 (3)	1.0702 (2)	0.4664 (2)	0.0401 (9)	
H24	0.5768	1.1255	0.4914	0.048*	
C25	0.4865 (3)	1.0608 (2)	0.4053 (2)	0.0362 (8)	
H25	0.4451	1.1094	0.3887	0.043*	
C26	0.2065 (2)	0.8593 (2)	0.46489 (19)	0.0284 (7)	
C27	0.2139 (3)	0.9414 (2)	0.5150 (2)	0.0452 (9)	
H27A	0.1483	0.9745	0.5086	0.068*	
H27B	0.2731	0.9762	0.4937	0.068*	
H27C	0.2251	0.9277	0.5756	0.068*	
C28	−0.0892 (3)	0.9341 (3)	0.3905 (3)	0.0584 (12)	
H28A	−0.0235	0.9626	0.4079	0.088*	
H28B	−0.1399	0.9362	0.4378	0.088*	
H28C	−0.1187	0.9641	0.3406	0.088*	
C29	0.1046 (3)	0.6183 (2)	−0.0140 (2)	0.0339 (8)	
H29A	0.0383	0.5980	−0.0393	0.051*	
H29B	0.1589	0.5735	−0.0212	0.051*	
H29C	0.1272	0.6718	−0.0427	0.051*	
C30	−0.0119 (3)	0.5751 (2)	0.3337 (2)	0.0311 (7)	
H30A	0.0555	0.5451	0.3248	0.047*	
H30B	−0.0696	0.5328	0.3315	0.047*	
H30C	−0.0115	0.6038	0.3897	0.047*	
C31	−0.2345 (2)	0.81935 (19)	0.2462 (2)	0.0284 (7)	
H31A	−0.3027	0.7887	0.2400	0.034*	
H31B	−0.2249	0.8348	0.3073	0.034*	
C32	−0.3153 (3)	0.9539 (2)	0.2150 (2)	0.0399 (8)	
H32A	−0.3834	0.9252	0.2058	0.060*	
H32B	−0.3104	1.0054	0.1783	0.060*	
H32C	−0.3092	0.9714	0.2751	0.060*	
C33	0.5015 (3)	0.6510 (2)	0.3335 (2)	0.0445 (9)	
H33A	0.4696	0.6603	0.3899	0.067*	
H33B	0.5785	0.6529	0.3386	0.067*	
H33C	0.4800	0.5941	0.3113	0.067*	
N1	−0.02662 (19)	0.63950 (15)	0.26699 (16)	0.0239 (5)	
O1	0.20493 (15)	0.87584 (12)	0.38022 (12)	0.0241 (4)	
O2	0.2048 (2)	0.78785 (15)	0.49642 (14)	0.0402 (6)	
O3	0.08940 (16)	0.63488 (13)	0.07442 (13)	0.0263 (5)	
O4	−0.27732 (15)	0.68993 (14)	0.12299 (15)	0.0316 (5)	
H4	−0.2766	0.6427	0.1499	0.047*	
O5	−0.23341 (17)	0.89599 (14)	0.19455 (15)	0.0349 (6)	
O6	−0.06838 (16)	0.84649 (14)	0.36913 (14)	0.0328 (5)	
O7	0.43716 (15)	0.76827 (15)	0.12268 (13)	0.0309 (5)	
H7	0.4897	0.7566	0.1534	0.046*	
O8	0.46752 (16)	0.71676 (14)	0.27663 (14)	0.0305 (5)	
O9	0.39220 (15)	0.89139 (13)	0.26615 (14)	0.0257 (5)	
O10	0.25977 (17)	0.66318 (13)	0.37478 (14)	0.0290 (5)	
H10A	0.2344	0.6755	0.4230	0.044*	
O11	0.32507 (17)	1.02599 (13)	0.27820 (16)	0.0349 (5)	
H2	0.037 (2)	0.8949 (9)	0.297 (2)	0.042*	

### Atomic displacement parameters (Å^2^)

**Table:**

	*U*^11^	*U*^22^	*U*^33^	*U*^12^	*U*^13^	*U*^23^
C1	0.0223 (14)	0.0175 (14)	0.0261 (15)	−0.0007 (12)	0.0000 (12)	0.0027 (12)
C2	0.0242 (15)	0.0225 (15)	0.0272 (16)	−0.0032 (12)	0.0023 (13)	−0.0019 (13)
C3	0.0241 (14)	0.0210 (14)	0.0204 (14)	−0.0024 (12)	0.0003 (12)	0.0007 (12)
C4	0.0231 (14)	0.0190 (14)	0.0207 (14)	−0.0019 (12)	−0.0004 (12)	−0.0012 (12)
C5	0.0216 (14)	0.0180 (14)	0.0261 (15)	−0.0037 (12)	−0.0013 (12)	0.0012 (12)
C6	0.0224 (14)	0.0220 (15)	0.0215 (15)	−0.0024 (12)	−0.0015 (12)	0.0018 (12)
C7	0.0203 (14)	0.0180 (14)	0.0239 (15)	−0.0014 (11)	−0.0029 (12)	−0.0007 (12)
C8	0.0251 (15)	0.0201 (15)	0.0227 (14)	0.0031 (12)	−0.0018 (12)	0.0023 (12)
C9	0.0263 (15)	0.0254 (16)	0.0251 (16)	−0.0045 (13)	−0.0068 (13)	−0.0003 (13)
C10	0.0192 (14)	0.0249 (15)	0.0283 (15)	−0.0032 (12)	−0.0032 (12)	0.0052 (13)
C11	0.0217 (14)	0.0211 (15)	0.0281 (15)	−0.0004 (12)	−0.0001 (12)	0.0029 (13)
C12	0.0231 (14)	0.0248 (15)	0.0282 (16)	−0.0053 (12)	−0.0001 (13)	0.0023 (13)
C13	0.0218 (14)	0.0198 (14)	0.0263 (16)	−0.0016 (12)	−0.0013 (12)	0.0010 (13)
C14	0.0273 (15)	0.0290 (16)	0.0263 (15)	−0.0029 (14)	0.0031 (13)	−0.0012 (13)
C15	0.0242 (14)	0.0255 (15)	0.0243 (15)	−0.0033 (13)	0.0022 (12)	0.0001 (13)
C16	0.0244 (15)	0.0248 (15)	0.0235 (15)	−0.0030 (13)	−0.0028 (12)	0.0058 (12)
C17	0.0230 (15)	0.0217 (15)	0.0311 (16)	−0.0013 (12)	0.0002 (13)	−0.0003 (13)
C18	0.0259 (15)	0.0225 (15)	0.0237 (15)	−0.0018 (13)	0.0000 (13)	0.0021 (12)
C19	0.0233 (15)	0.0203 (15)	0.0337 (18)	−0.0055 (13)	0.0016 (13)	0.0034 (13)
C20	0.0217 (14)	0.0266 (15)	0.0267 (16)	−0.0045 (13)	0.0035 (13)	0.0004 (13)
C21	0.0336 (17)	0.0256 (16)	0.0307 (18)	−0.0019 (14)	−0.0003 (14)	0.0026 (14)
C22	0.0373 (18)	0.0350 (19)	0.0343 (19)	0.0011 (16)	−0.0072 (15)	0.0090 (16)
C23	0.0339 (18)	0.047 (2)	0.0284 (18)	−0.0087 (16)	−0.0056 (15)	0.0040 (16)
C24	0.0358 (19)	0.036 (2)	0.049 (2)	−0.0075 (16)	−0.0028 (17)	−0.0144 (17)
C25	0.0302 (17)	0.0269 (17)	0.051 (2)	0.0008 (14)	−0.0034 (16)	−0.0027 (16)
C26	0.0288 (16)	0.0304 (18)	0.0260 (16)	−0.0039 (14)	0.0002 (14)	−0.0031 (14)
C27	0.064 (3)	0.035 (2)	0.036 (2)	−0.0062 (19)	−0.0018 (19)	−0.0077 (16)
C28	0.043 (2)	0.050 (2)	0.082 (3)	−0.0042 (19)	0.019 (2)	−0.034 (2)
C29	0.0347 (17)	0.0335 (18)	0.0335 (18)	0.0068 (15)	0.0028 (15)	−0.0042 (15)
C30	0.0338 (17)	0.0234 (16)	0.0362 (18)	−0.0041 (14)	−0.0038 (15)	0.0080 (14)
C31	0.0241 (15)	0.0279 (16)	0.0332 (17)	−0.0026 (13)	0.0009 (13)	0.0014 (14)
C32	0.041 (2)	0.0364 (19)	0.043 (2)	0.0109 (16)	0.0066 (17)	0.0022 (17)
C33	0.0345 (19)	0.045 (2)	0.054 (2)	0.0089 (17)	−0.0026 (17)	0.0134 (19)
N1	0.0250 (13)	0.0197 (12)	0.0269 (13)	−0.0043 (11)	−0.0018 (11)	0.0047 (11)
O1	0.0270 (10)	0.0215 (10)	0.0238 (11)	−0.0029 (9)	−0.0012 (9)	−0.0031 (9)
O2	0.0527 (15)	0.0367 (14)	0.0313 (12)	−0.0038 (12)	0.0017 (11)	0.0005 (11)
O3	0.0290 (11)	0.0230 (11)	0.0270 (11)	0.0031 (9)	−0.0036 (9)	−0.0029 (9)
O4	0.0239 (11)	0.0305 (12)	0.0405 (13)	−0.0063 (9)	−0.0056 (10)	0.0057 (11)
O5	0.0348 (12)	0.0273 (12)	0.0424 (14)	0.0098 (10)	0.0107 (11)	0.0074 (10)
O6	0.0293 (11)	0.0338 (12)	0.0354 (12)	−0.0005 (10)	0.0084 (10)	−0.0081 (11)
O7	0.0232 (10)	0.0401 (13)	0.0293 (11)	−0.0012 (10)	0.0048 (9)	0.0014 (11)
O8	0.0248 (11)	0.0300 (12)	0.0367 (12)	0.0019 (9)	0.0003 (10)	0.0067 (10)
O9	0.0231 (10)	0.0224 (10)	0.0318 (12)	−0.0035 (9)	−0.0030 (9)	0.0003 (9)
O10	0.0326 (11)	0.0249 (11)	0.0297 (12)	−0.0004 (9)	0.0023 (10)	0.0080 (10)
O11	0.0287 (12)	0.0229 (11)	0.0532 (15)	0.0023 (10)	−0.0106 (11)	0.0011 (11)

### Geometric parameters (Å, °)

**Table:**

C1—C11	1.547 (4)	C18—O10	1.422 (3)
C1—C7	1.570 (4)	C18—H18	1.0000
C1—C2	1.570 (4)	C19—O11	1.206 (3)
C1—H1	1.0000	C19—O9	1.339 (3)
C2—O6	1.424 (4)	C19—C20	1.489 (4)
C2—C3	1.556 (4)	C20—C25	1.387 (4)
C2—H2	0.970 (16)	C20—C21	1.391 (4)
C3—C4	1.533 (4)	C21—C22	1.379 (4)
C3—C13	1.551 (4)	C21—H21	0.9500
C3—H3	1.0000	C22—C23	1.376 (5)
C4—O1	1.488 (3)	C22—H22	0.9500
C4—C5	1.539 (4)	C23—C24	1.377 (5)
C4—C18	1.560 (4)	C23—H23	0.9500
C5—C16	1.522 (4)	C24—C25	1.380 (5)
C5—C6	1.562 (4)	C24—H24	0.9500
C5—H5	1.0000	C25—H25	0.9500
C6—C7	1.559 (4)	C26—O2	1.204 (4)
C6—C14	1.561 (4)	C26—O1	1.346 (4)
C6—H6	1.0000	C26—C27	1.490 (4)
C7—C13	1.533 (4)	C27—H27A	0.9800
C7—C8	1.545 (4)	C27—H27B	0.9800
C8—O3	1.431 (3)	C27—H27C	0.9800
C8—C9	1.521 (4)	C28—O6	1.413 (4)
C8—H8	1.0000	C28—H28A	0.9800
C9—C10	1.506 (4)	C28—H28B	0.9800
C9—H9A	0.9900	C28—H28C	0.9800
C9—H9B	0.9900	C29—O3	1.417 (4)
C10—O4	1.435 (3)	C29—H29A	0.9800
C10—C11	1.540 (4)	C29—H29B	0.9800
C10—H10	1.0000	C29—H29C	0.9800
C11—C31	1.534 (4)	C30—N1	1.449 (4)
C11—C12	1.540 (4)	C30—H30A	0.9800
C12—N1	1.480 (4)	C30—H30B	0.9800
C12—H12A	0.9900	C30—H30C	0.9800
C12—H12B	0.9900	C31—O5	1.429 (4)
C13—N1	1.464 (4)	C31—H31A	0.9900
C13—H13	1.0000	C31—H31B	0.9900
C14—C15	1.526 (4)	C32—O5	1.405 (4)
C14—H14A	0.9900	C32—H32A	0.9800
C14—H14B	0.9900	C32—H32B	0.9800
C15—O7	1.420 (3)	C32—H32C	0.9800
C15—C16	1.516 (4)	C33—O8	1.413 (4)
C15—C17	1.539 (4)	C33—H33A	0.9800
C16—O9	1.437 (3)	C33—H33B	0.9800
C16—H16	1.0000	C33—H33C	0.9800
C17—O8	1.429 (3)	O4—H4	0.8400
C17—C18	1.550 (4)	O7—H7	0.8400
C17—H17	1.0000	O10—H10A	0.8400
			
C11—C1—C7	110.0 (2)	O9—C16—H16	110.3
C11—C1—C2	112.6 (2)	C15—C16—H16	110.3
C7—C1—C2	102.1 (2)	C5—C16—H16	110.3
C11—C1—H1	110.6	O8—C17—C15	106.6 (2)
C7—C1—H1	110.6	O8—C17—C18	109.3 (2)
C2—C1—H1	110.6	C15—C17—C18	114.9 (2)
O6—C2—C3	109.5 (2)	O8—C17—H17	108.6
O6—C2—C1	117.6 (2)	C15—C17—H17	108.6
C3—C2—C1	104.7 (2)	C18—C17—H17	108.6
O6—C2—H2	104 (2)	O10—C18—C17	107.3 (2)
C3—C2—H2	113 (2)	O10—C18—C4	112.6 (2)
C1—C2—H2	109 (2)	C17—C18—C4	117.6 (2)
C4—C3—C13	112.2 (2)	O10—C18—H18	106.2
C4—C3—C2	107.8 (2)	C17—C18—H18	106.2
C13—C3—C2	104.1 (2)	C4—C18—H18	106.2
C4—C3—H3	110.8	O11—C19—O9	123.9 (3)
C13—C3—H3	110.8	O11—C19—C20	126.5 (3)
C2—C3—H3	110.8	O9—C19—C20	109.7 (2)
O1—C4—C3	108.3 (2)	C25—C20—C21	119.7 (3)
O1—C4—C5	101.4 (2)	C25—C20—C19	119.5 (3)
C3—C4—C5	108.1 (2)	C21—C20—C19	120.8 (3)
O1—C4—C18	107.7 (2)	C22—C21—C20	119.7 (3)
C3—C4—C18	116.6 (2)	C22—C21—H21	120.1
C5—C4—C18	113.7 (2)	C20—C21—H21	120.1
C16—C5—C4	112.2 (2)	C23—C22—C21	120.5 (3)
C16—C5—C6	101.9 (2)	C23—C22—H22	119.8
C4—C5—C6	111.7 (2)	C21—C22—H22	119.8
C16—C5—H5	110.3	C22—C23—C24	119.9 (3)
C4—C5—H5	110.3	C22—C23—H23	120.1
C6—C5—H5	110.3	C24—C23—H23	120.1
C7—C6—C14	115.5 (2)	C23—C24—C25	120.4 (3)
C7—C6—C5	117.3 (2)	C23—C24—H24	119.8
C14—C6—C5	102.8 (2)	C25—C24—H24	119.8
C7—C6—H6	106.8	C24—C25—C20	119.8 (3)
C14—C6—H6	106.8	C24—C25—H25	120.1
C5—C6—H6	106.8	C20—C25—H25	120.1
C13—C7—C8	116.2 (2)	O2—C26—O1	125.0 (3)
C13—C7—C6	109.1 (2)	O2—C26—C27	124.1 (3)
C8—C7—C6	108.0 (2)	O1—C26—C27	110.8 (3)
C13—C7—C1	98.5 (2)	C26—C27—H27A	109.5
C8—C7—C1	112.4 (2)	C26—C27—H27B	109.5
C6—C7—C1	112.5 (2)	H27A—C27—H27B	109.5
O3—C8—C9	107.7 (2)	C26—C27—H27C	109.5
O3—C8—C7	109.5 (2)	H27A—C27—H27C	109.5
C9—C8—C7	116.4 (2)	H27B—C27—H27C	109.5
O3—C8—H8	107.6	O6—C28—H28A	109.5
C9—C8—H8	107.6	O6—C28—H28B	109.5
C7—C8—H8	107.6	H28A—C28—H28B	109.5
C10—C9—C8	112.7 (2)	O6—C28—H28C	109.5
C10—C9—H9A	109.0	H28A—C28—H28C	109.5
C8—C9—H9A	109.0	H28B—C28—H28C	109.5
C10—C9—H9B	109.0	O3—C29—H29A	109.5
C8—C9—H9B	109.0	O3—C29—H29B	109.5
H9A—C9—H9B	107.8	H29A—C29—H29B	109.5
O4—C10—C9	110.3 (2)	O3—C29—H29C	109.5
O4—C10—C11	113.0 (2)	H29A—C29—H29C	109.5
C9—C10—C11	112.1 (2)	H29B—C29—H29C	109.5
O4—C10—H10	107.0	N1—C30—H30A	109.5
C9—C10—H10	107.0	N1—C30—H30B	109.5
C11—C10—H10	107.0	H30A—C30—H30B	109.5
C31—C11—C10	106.4 (2)	N1—C30—H30C	109.5
C31—C11—C12	110.0 (2)	H30A—C30—H30C	109.5
C10—C11—C12	112.1 (2)	H30B—C30—H30C	109.5
C31—C11—C1	111.3 (2)	O5—C31—C11	107.6 (2)
C10—C11—C1	109.1 (2)	O5—C31—H31A	110.2
C12—C11—C1	108.0 (2)	C11—C31—H31A	110.2
N1—C12—C11	112.9 (2)	O5—C31—H31B	110.2
N1—C12—H12A	109.0	C11—C31—H31B	110.2
C11—C12—H12A	109.0	H31A—C31—H31B	108.5
N1—C12—H12B	109.0	O5—C32—H32A	109.5
C11—C12—H12B	109.0	O5—C32—H32B	109.5
H12A—C12—H12B	107.8	H32A—C32—H32B	109.5
N1—C13—C7	109.9 (2)	O5—C32—H32C	109.5
N1—C13—C3	115.7 (2)	H32A—C32—H32C	109.5
C7—C13—C3	100.3 (2)	H32B—C32—H32C	109.5
N1—C13—H13	110.2	O8—C33—H33A	109.5
C7—C13—H13	110.2	O8—C33—H33B	109.5
C3—C13—H13	110.2	H33A—C33—H33B	109.5
C15—C14—C6	107.1 (2)	O8—C33—H33C	109.5
C15—C14—H14A	110.3	H33A—C33—H33C	109.5
C6—C14—H14A	110.3	H33B—C33—H33C	109.5
C15—C14—H14B	110.3	C30—N1—C13	113.1 (2)
C6—C14—H14B	110.3	C30—N1—C12	110.1 (2)
H14A—C14—H14B	108.5	C13—N1—C12	116.4 (2)
O7—C15—C16	113.1 (2)	C26—O1—C4	121.0 (2)
O7—C15—C14	110.1 (2)	C29—O3—C8	113.7 (2)
C16—C15—C14	102.2 (2)	C10—O4—H4	109.5
O7—C15—C17	110.1 (2)	C32—O5—C31	112.8 (2)
C16—C15—C17	110.4 (2)	C28—O6—C2	112.9 (3)
C14—C15—C17	110.8 (2)	C15—O7—H7	109.5
O9—C16—C15	108.1 (2)	C33—O8—C17	115.5 (2)
O9—C16—C5	115.7 (2)	C19—O9—C16	120.7 (2)
C15—C16—C5	101.9 (2)	C18—O10—H10A	109.5
			
C11—C1—C2—O6	−26.0 (4)	C2—C3—C13—C7	40.7 (3)
C7—C1—C2—O6	−143.9 (2)	C7—C6—C14—C15	−133.1 (2)
C11—C1—C2—C3	95.8 (3)	C5—C6—C14—C15	−4.0 (3)
C7—C1—C2—C3	−22.2 (3)	C6—C14—C15—O7	−145.6 (2)
O6—C2—C3—C4	−124.5 (2)	C6—C14—C15—C16	−25.2 (3)
C1—C2—C3—C4	108.5 (2)	C6—C14—C15—C17	92.4 (3)
O6—C2—C3—C13	116.1 (2)	O7—C15—C16—O9	−73.8 (3)
C1—C2—C3—C13	−10.9 (3)	C14—C15—C16—O9	167.8 (2)
C13—C3—C4—O1	175.3 (2)	C17—C15—C16—O9	50.0 (3)
C2—C3—C4—O1	61.2 (3)	O7—C15—C16—C5	163.8 (2)
C13—C3—C4—C5	66.2 (3)	C14—C15—C16—C5	45.5 (3)
C2—C3—C4—C5	−47.9 (3)	C17—C15—C16—C5	−72.4 (3)
C13—C3—C4—C18	−63.2 (3)	C4—C5—C16—O9	−45.8 (3)
C2—C3—C4—C18	−177.4 (2)	C6—C5—C16—O9	−165.3 (2)
O1—C4—C5—C16	89.2 (3)	C4—C5—C16—C15	71.2 (3)
C3—C4—C5—C16	−157.1 (2)	C6—C5—C16—C15	−48.4 (3)
C18—C4—C5—C16	−26.0 (3)	O7—C15—C17—O8	33.8 (3)
O1—C4—C5—C6	−157.2 (2)	C16—C15—C17—O8	−91.8 (3)
C3—C4—C5—C6	−43.5 (3)	C14—C15—C17—O8	155.8 (2)
C18—C4—C5—C6	87.6 (3)	O7—C15—C17—C18	155.1 (2)
C16—C5—C6—C7	159.5 (2)	C16—C15—C17—C18	29.5 (3)
C4—C5—C6—C7	39.7 (3)	C14—C15—C17—C18	−82.9 (3)
C16—C5—C6—C14	31.6 (3)	O8—C17—C18—O10	−95.0 (3)
C4—C5—C6—C14	−88.3 (3)	C15—C17—C18—O10	145.2 (2)
C14—C6—C7—C13	69.8 (3)	O8—C17—C18—C4	136.9 (3)
C5—C6—C7—C13	−51.7 (3)	C15—C17—C18—C4	17.0 (4)
C14—C6—C7—C8	−57.4 (3)	O1—C4—C18—O10	104.2 (3)
C5—C6—C7—C8	−178.9 (2)	C3—C4—C18—O10	−17.5 (3)
C14—C6—C7—C1	178.0 (2)	C5—C4—C18—O10	−144.3 (2)
C5—C6—C7—C1	56.5 (3)	O1—C4—C18—C17	−130.2 (2)
C11—C1—C7—C13	−72.6 (3)	C3—C4—C18—C17	108.0 (3)
C2—C1—C7—C13	47.1 (2)	C5—C4—C18—C17	−18.7 (3)
C11—C1—C7—C8	50.4 (3)	O11—C19—C20—C25	−6.9 (5)
C2—C1—C7—C8	170.1 (2)	O9—C19—C20—C25	172.2 (3)
C11—C1—C7—C6	172.5 (2)	O11—C19—C20—C21	174.7 (3)
C2—C1—C7—C6	−67.8 (3)	O9—C19—C20—C21	−6.2 (4)
C13—C7—C8—O3	−52.6 (3)	C25—C20—C21—C22	0.2 (5)
C6—C7—C8—O3	70.3 (3)	C19—C20—C21—C22	178.6 (3)
C1—C7—C8—O3	−165.0 (2)	C20—C21—C22—C23	0.1 (5)
C13—C7—C8—C9	69.9 (3)	C21—C22—C23—C24	−0.7 (5)
C6—C7—C8—C9	−167.2 (2)	C22—C23—C24—C25	0.9 (5)
C1—C7—C8—C9	−42.5 (3)	C23—C24—C25—C20	−0.6 (5)
O3—C8—C9—C10	166.8 (2)	C21—C20—C25—C24	0.0 (5)
C7—C8—C9—C10	43.4 (3)	C19—C20—C25—C24	−178.4 (3)
C8—C9—C10—O4	−179.6 (2)	C10—C11—C31—O5	−68.7 (3)
C8—C9—C10—C11	−52.7 (3)	C12—C11—C31—O5	169.7 (2)
O4—C10—C11—C31	−52.4 (3)	C1—C11—C31—O5	50.1 (3)
C9—C10—C11—C31	−177.8 (2)	C7—C13—N1—C30	172.1 (2)
O4—C10—C11—C12	67.9 (3)	C3—C13—N1—C30	−75.2 (3)
C9—C10—C11—C12	−57.5 (3)	C7—C13—N1—C12	−59.0 (3)
O4—C10—C11—C1	−172.6 (2)	C3—C13—N1—C12	53.7 (3)
C9—C10—C11—C1	62.0 (3)	C11—C12—N1—C30	174.3 (2)
C7—C1—C11—C31	−177.0 (2)	C11—C12—N1—C13	44.0 (3)
C2—C1—C11—C31	69.9 (3)	O2—C26—O1—C4	2.3 (5)
C7—C1—C11—C10	−59.9 (3)	C27—C26—O1—C4	−179.4 (3)
C2—C1—C11—C10	−173.0 (2)	C3—C4—O1—C26	69.5 (3)
C7—C1—C11—C12	62.2 (3)	C5—C4—O1—C26	−176.9 (2)
C2—C1—C11—C12	−51.0 (3)	C18—C4—O1—C26	−57.3 (3)
C31—C11—C12—N1	−165.4 (2)	C9—C8—O3—C29	84.5 (3)
C10—C11—C12—N1	76.4 (3)	C7—C8—O3—C29	−148.0 (2)
C1—C11—C12—N1	−43.8 (3)	C11—C31—O5—C32	178.3 (3)
C8—C7—C13—N1	−51.9 (3)	C3—C2—O6—C28	136.2 (3)
C6—C7—C13—N1	−174.2 (2)	C1—C2—O6—C28	−104.5 (3)
C1—C7—C13—N1	68.3 (3)	C15—C17—O8—C33	−164.5 (3)
C8—C7—C13—C3	−174.2 (2)	C18—C17—O8—C33	70.7 (3)
C6—C7—C13—C3	63.4 (3)	O11—C19—O9—C16	−3.7 (4)
C1—C7—C13—C3	−54.0 (2)	C20—C19—O9—C16	177.3 (2)
C4—C3—C13—N1	166.2 (2)	C15—C16—O9—C19	179.1 (2)
C2—C3—C13—N1	−77.4 (3)	C5—C16—O9—C19	−67.5 (3)
C4—C3—C13—C7	−75.7 (3)		

### Hydrogen-bond geometry (Å, °)

**Table:**

*D*—H···*A*	*D*—H	H···*A*	*D*···*A*	*D*—H···*A*
O10—H10A···O2	0.84	2.11	2.788 (3)	138
O7—H7···O8	0.84	2.04	2.560 (3)	120
O4—H4···O11^i^	0.84	2.20	3.018 (3)	163

Symmetry codes: (i) −*x*, *y*−1/2, −*z*+1/2.
